# Digital literacy and subjective happiness of low-income groups: Evidence from rural China

**DOI:** 10.3389/fpsyg.2022.1045187

**Published:** 2022-11-23

**Authors:** Jie Wang, Chang Liu, Zhijian Cai

**Affiliations:** College of Economics and Management, Nanjing Forestry University, Nanjing, China

**Keywords:** digital technology, digital economy, relative poverty, subjective happiness, vulnerable groups

## Abstract

Improvements of the happiness of the rural population are an essential sign of the effectiveness of relative poverty governance. In the context of today’s digital economy, assessing the relationship between digital literacy and the subjective happiness of rural low-income groups is of great practicality. Based on data from China Family Panel Studies, the effect of digital literacy on the subjective well-being of rural low-income groups was empirically tested. A significant happiness effect of digital literacy on rural low-income groups was found. Digital literacy promotes the subjective happiness of rural low-income groups through income increase and consumption growth effects. The observed happiness effect is heterogeneous among different characteristic groups, and digital literacy significantly positively impacts the subjective happiness of rural low-income groups. Decomposition of subjective happiness into life satisfaction and job satisfaction shows that digital literacy significantly positively affects the job and life satisfaction of rural low-income groups. This paper demonstrates that digital literacy induces a practical happiness effect. To further strengthen the subjective welfare effect of digital literacy in the construction of digital villages, the government should focus on cultivating digital literacy among low-income groups from the demand side. The construction of digital infrastructure should be actively promoted from the supply side.

## Introduction

Over recent years, China has achieved unprecedented poverty eradication, and a total of 55.75 million rural poor people have been completely lifted out of poverty. The lessons learned contribute an important solution to poverty alleviation in developing countries. With the completion of the task of eradicating absolute poverty in China according to current standards, the focus of today’s post-poverty alleviation era will shift to alleviating relative poverty with unbalanced and insufficient development (Wan et al., 2021). How to manage relative poverty has become a new issue for both the government and academia. With the further social and economic development, the meaning of individual welfare is not limited to objective economic welfare but also embodies subjective happiness. Subjective happiness has gradually become an essential expression of individual welfare (Oswald and Wu, 2010). Unlike absolute poverty management, relative poverty management addresses the relative shortage of welfare at the economic level and alleviates the relative lack of subjective welfare. The Chinese government has long attached great importance to the happiness of its population. Policy documents issued by the State Council have repeatedly emphasized the importance of improving the happiness of rural residents. At the stage of relative poverty governance in China, improving the happiness of rural residents has become an important yardstick for measuring effective poverty governance (Qiu et al., 2021).

With the construction of information infrastructure, service platforms, and resource systems on the supply side, digital technologies are gradually being integrated into different areas of rural production, life, ecology, and governance. Digital technology applications provide rural residents with diversified opportunities and tend to equalize access and benefit. However, in economic activities, rural residents do not benefit equally from digital development outcomes (Scheerder et al., 2017). Differences in the digital literacy of individuals may cause significant differences in their level of participation and the benefit they obtain in the digital economy. Digital literacy represents an individual’s attitude or ability to properly use digital tools and devices, utilize digital resources, learn new knowledge, and interact socially in the emerging digital environment (Martin and Grudziecki, 2006; Eshet, 2012). It has been shown that based on governmental provision of various Internet infrastructure types, disadvantaged groups in rural areas can lower the financial threshold for obtaining digital access. However, these disadvantaged groups must first acquire a certain level of digital literacy to better access economic opportunities and social rights (Akerman et al., 2015). Thus, an important link may exist between digital literacy and the subjective happiness of rural residents, which may be a key means for enhancing happiness.

For rural residents, two levels of digital technology adoption exist in digital technology accessibility, reflecting differences in access and usage of digital technology, as well as differences in digital literacy (Scheerder et al., 2017). Previous literature has discussed the impact of the gap to access digital technology on subjective happiness. Theoretically, the adoption of digital technology may impact people’s subjective happiness through channels such as reducing the cost of access to information, providing leisure and entertainment activities, increasing social activities, and promoting consumption on online platforms (Cilesiz, 2009; Purcell et al., 2013; Campante et al., 2018). DiMaggio and Bonikowski (2008) found that digital technology applications can promote happiness by indirectly reducing residents’ perceptions of the importance of pursuing material income. The happiness effect is stronger for rural residents than for urban residents in different regions; it is also stronger for residents of central and western regions compared with residents of eastern regions (Long et al., 2020). In addition to regional differences, differences in individual characteristics also affect the happiness effect when adopting digital technology. It has been shown that Internet use suppresses happiness in older people with low literacy levels while it facilitates happiness in older people with high literacy levels (Yang et al., 2021). It has also been argued that digital technology applications may undermine subjective happiness. Excessive information references on Internet platforms can cause people to easily engage in comparisons with others, causing them to underestimate their own economic and social status (Lohmann, 2015). Individuals’ excessive reliance and use of the Internet can cause atrophy of offline emotional interactions and reduce people’s interest in participating in social activities, which reduces happiness (Huang, 2010; Sabatini and Sarracino, 2017).

Overall, the existing literature has paid little attention to the relationship between the usage of digital technology and happiness, which is hidden within the accessibility of digital technology. With the improvement of digital infrastructure and its wider availability, the gap in digital technology accessibility continues to narrow^1^. Soon, digital access will become equal, and the level of participating in and benefitting from the digital economy by rural residents will depend mainly on their digital literacy (Scheerder et al., 2017). Feasibility theory suggests that the prerequisite for individuals to engage in functional activities is the availability of corresponding capabilities that make this engagement feasible (Sen, 1999). In the context of the rapid development of the digital economy, the feasible digital literacy ability determines whether individuals can participate in digital-related production and business activities adaptively, rationally, and effectively (Akerman et al., 2015). To test whether increased digital literacy benefits rural populations and whether such an effect varies across contexts and populations, more micro-empirical results are needed. In terms of the research subjects, the existing literature takes urban groups or general rural groups as the research subjects, and less often focuses on rural low-income groups—a socially marginalized group. Relative deprivation theory suggests that individuals who find themselves at a disadvantage compared to others in the same life situation will experience a sense of deprivation (Townsend, 1979). This sense strongly impairs the happiness of such groups. Low-income groups are more likely to be relatively deprived of their rights because of both income and ability poverty. Therefore, low-income groups experience a greater sense of deprivation than other groups. In the governance of relative poverty, the characteristics of low-income group support projects are their long cycle and high complexity (Wan et al., 2021). At this stage, the focus of support and adaptive governance for low-income groups should not only be the establishment of a long-term mechanism to increase income and prevent a return to poverty, but also to pay attention to the subjective welfare of low-income groups.

This paper focuses on the impact of digital literacy on the subjective happiness of the rural population, especially addressing the subjective happiness of rural low-income groups. Based on this study, scientific suggestions for relative poverty governance are proposed from the perspective of subjective welfare. The marginal contributions of this paper are: first, the innovation of research perspective. Referring to the mindsponge theory, we construct an analytical framework of digital literacy affecting subjective happiness from an information perspective, which enriches the research results on the antecedents of happiness to a certain extent. Second, the innovation of the research object. The research object is aimed at the rural low-income group, a socially marginalized group. Third, the expansion of the connotation of happiness. This paper decomposes happiness into life satisfaction and job satisfaction, and analyzes the influence of digital literacy on the work and life satisfaction of rural low-income groups, which enriches the connotation of subjective happiness.

## Theoretical framework and hypotheses

### Determinants of subjective happiness

“Happiness” is a subjective cognitive evaluation and emotional experience of an individual’s real-life state that ranges on a continuum from positive to negative and is a subjective reflection at the psychological level (Diener et al., 2003). Previous studies have identified income as an essential factor affecting the happiness of rural low-income groups (Easterlin et al., 2012; Jebb et al., 2018). Academics usually analyze the relationship between income and happiness from either an absolute or a relative perspective. In rural low-income groups, subjective happiness is affected by both absolute and relative incomes. First, individual absolute income can increase the happiness of low-income groups by relaxing household budget constraints (Easterlin, 2001). Second, in situations where it is challenging to increase absolute income, narrowing the relative income gap can alleviate the relative deprivation low-income groups experience and can thus enhance subjective happiness (Ding et al., 2021). According to relative deprivation theory, the essence of relative deprivation is the sense of deprivation that arises when individuals find themselves at a disadvantage compared with others in their living situation; relative deprivation causes a significant loss of happiness in such groups (Runciman, 1966). For rural low-income groups, relative deprivation is not only relative “poverty” in economic terms but also relative “deprivation” in terms of access to economic and social rights (such as education, health services, and housing) caused by social disadvantages (Sen, 1999).

From a consumption perspective, the level and structure of consumption reflect the outcome of the individual’s realization of the implementation of their rights in the real world, e.g., the right to medical services, education, or housing. Compared to income indicators, the consumption structure provides a more comprehensive representation of individual resource endowments as well as the implementation of economic and social rights. Traditional economic theory suggests that utility is directly derived from consumption. Therefore, changes in the consumption structure exert an important impact on the subjective happiness of rural low-income groups. According to individual needs, consumption structures have been classified into consumption of basic goods, conspicuous consumption, material consumption, and experiential consumption (Zimmermann, 2014). In the Chinese context, the types of subsistence consumption have been classified into subsistence consumption, developmental consumption, and enjoyment consumption (Qi and Liu, 2020). Among these forms of consumption, subsistence consumption satisfies physiological needs, expressed as the demand for food and housing conditions. The purpose of developmental consumption is to meet individuals’ needs for the pursuit of better developmental goals, including education and medical services. Enjoyment-based consumption represents consumption with the goal to satisfy the desire for enjoyment, such as entertainment and cultural expenditures. Regarding the relationship between consumption and happiness, it has been shown that both the absolute and relative values of household consumption contribute to the enhancement of happiness (Weidman and Dunn, 2016; Tsurumi et al., 2021). Among different types of consumption, expenditures for basic food, equipment, supplies, housing, and medical services significantly positively affect subjective happiness (Borrero Caldas, 2010). For low-income groups, based on the ability of individuals to satisfy their survival needs, the right to pursue further development and fulfil enjoyment desires can greatly enhance subjective happiness.

### The effect of digital literacy on the subjective happiness of rural low-income groups

With the implementation of a series of policies (such as “broadband China”), the construction and improvement of China’s information infrastructure have accelerated. The application of digital technologies such as big data, the Internet of Things, and artificial intelligence in the production sector has led to the digital development of rural industries, embedding digital technologies in all stages and sectors of production. Currently, digital technology has derived multiple functions for information interaction, such as digital learning and work, participation in business transactions, and social interaction (Nishijima et al., 2017). According to the mindsponge theory, as human minds are information processors, digital literacy can be understood as the mind’s ability to process information related to digital issues, ranging from information absorption, evaluation, filtering, and optimization (Eshet, 2012; Vuong et al., 2022). Subsequently, better digital literacy will lead to better outcomes of mental processes related to digital content (e.g., decision-making and behaviors). Meanwhile, although the mechanism behind subjective happiness is still not clearly defined, achieving fulfilment is one of the major determinants of subjective happiness. Therefore, with better digital literacy, rural low-income groups are more likely to have access to and capitalize on the information on the Internet for achieving fulfilment, such as increased income and consumption, which will result in higher subjective happiness.

#### Income effects of digital literacy

The absolute advantage of digital technology in information dissemination and employment effects provides the basis for increasing the absolute income and alleviating income inequality (Ma et al., 2020). In agricultural production, digital literacy allows individuals to exploit the information effect of digital technology more effectively. Consequently, market information can be obtained at a lower information-seeking cost, which helps to improve the market effect of agricultural products and increases agricultural income (Aker, 2010). Digital literacy also contributes to human capital accumulation. Digitally literate low-income groups learn by searching for information and resources on digital platforms (e.g., searching for instructional videos and shared experiences), thus effectively reducing the cost of the acquisition of knowledge and information for individuals (Castellacci and Tveito, 2018). In the labor market, digital literacy can help low-income groups to better use digital platforms when searching for employment information and achieve mobility from agricultural to non-agricultural occupations to increase income from wages (Kuhn and Mansour, 2014). In addition, digital literacy helps to absorb and develop information. Based on the searched market information, digitally literate low-income groups can accurately develop information and grasp market needs to develop new business opportunities, which can help them to join rural entrepreneurial activities (Dettling, 2017). The essence of income “poverty” is ability “distress,” and digital literacy, as a form of human capital, can create posterior advantages for low-income groups, thus narrowing the relative income gap and enhancing individuals’ subjective happiness (Philip et al., 2017). Shan et al. (2022) found that the elasticities of the effect of digital literacy on low- and middle-income groups are much higher than the elasticity of high-income groups. This reflects the characteristics of a decreasing marginal effect rather than an increasing scale.

#### The consumption effect of digital literacy

The imperfection of rural road transport facilities limits the range of products and services available in the offline markets of rural areas. Digital technology has overcome the barriers of information dissemination, increased the circulation of urban and rural goods, and expanded the scope of rural markets (Oshota, 2019). Digitally literate rural low-income groups can fulfil material consumption from digital shopping platforms, such as consuming specialty products or other livelihood-friendly products that are not available in their region (Pierrakis and Collins, 2013). While individuals may simultaneously have multiple needs, the dominant need exerts the greatest motivational effect on individual happiness. Survival-oriented consumption is the main aspect of the consumption structure of rural low-income groups, for whom the growth of survival-oriented consumption generates great life satisfaction (Holder, 2019). Second, the digital economy uses information as a factor of production. Digital literacy helps individuals to overcome the traditional model of mainly offline learning. Digitally literate individuals seek consumable educational information and resources from digital platforms, which enhances the sense of achievement and happiness in human capital accumulation (Castellacci and Tveito, 2018). Along with the gradual diversification and refinement of the functions and services of online platforms, the cost of information dissemination between doctors and patients has been reduced. For example, smart health services on digital platforms, especially Internet-based online medical services built by hospitals, have reduced the commuting time to medical services and the economic cost of accessing medical services (Nishijima et al., 2017). Digital literacy enhancement helps rural low-income groups to use online healthcare services thus alleviating the sense of relative deprivation. Digital literacy enhancement helps rural low-income groups to use online healthcare services, thus alleviating the relative deprivation of healthcare services. Finally, digital literacy helps rural low-income groups improve their cognitive ability and overcome the inherent concept of “materialism over culture,” thus forming diversified consumption needs (An et al., 2022). The income-increasing effect of digital literacy can also help rural low-income groups to better satisfy their hedonic consumption needs and thus increase their life satisfaction.

Accordingly, the following two hypotheses are proposed:

*Hypothesis 1*: Digital literacy has a significant happiness effect; the higher the digital literacy, the higher the subjective happiness of the rural low-income group.

*Hypothesis 2*: Digital literacy enhancement contributes to the subjective happiness of rural low-income groups through the income effect and consumption effects.

## Study design

### Empirical model

The following econometric model is constructed to assess the relationship between digital literacy and subjective happiness of rural low-income groups.


(1) $${\mathrm{Happiness}}_{i}=\alpha +\beta_{1}{\mathrm{Digital}}_{i}+\beta_{2}{\mathrm{Personality}}_{i}+\sum \beta_{3}X_{i}+\varepsilon_{i}$$

Where ${\mathrm{Happiness}}_{i}$ indicates the subjective happiness of rural low-income individuals. $\;{\mathrm{Digital}}_{i}$ denotes the digital literacy of rural low-income individuals, obtained through different dimensions of digital attitudes and digital abilities. $\;{\mathrm{Personality}}_{i}$ reflects the individuals’ personality traits, which can be extroversion, dutifulness, affinity, openness, and emotional stability. $\;X_{i}$ includes other individual and family characteristics such as age, gender, education level, and family size. $\varepsilon_{i}$ denotes the random error term. In addition, village level fixed effects are controlled.

Following the basic model setup described above, in the baseline regression section, ordinary least squares (ols) and ordered probit (oprobit) are adopted for parameter estimation.

Digital literacy is essentially an individual’s ability to use digital technology, and endogeneity problems may emerge in the process of causal identification, leading to biased estimations. In this paper, there are two types of endogeneity problems: first, the omission of variables, i.e., certain important variables that affect both digital literacy and subjective happiness may have been omitted from the baseline regression model. Second, reverse causality, i.e., individuals’ subjective happiness affects their digital literacy level and vice versa. For example, individuals may be more likely to learn and use digital technology and thus accumulate digital literacy when they feel positive emotions of happiness.

To mitigate endogeneity issues as much as possible, hard-to-observe personality traits as well as individual and family characteristics are controlled for in the baseline regression model. At the same time, an instrumental variable approach is used for parameter estimation. Drawing on existing literature (Deng et al., 2019; Ma et al., 2020), in this paper, individuals’ understanding of the importance of digital technology is selected as an instrumental variable for digital literacy. From the perspective of relevance, individuals who value digital technology may put it into practice behaviorally and thus develop digital literacy. From the perspective of exogeneity, the importance individuals place on digital technology is indirectly influenced by individual digital literacy even if it may impact their subjective happiness. Based on the above, this paper argues that the importance individuals place on digital technology is a reasonable instrumental variable.

This paper uses two-stage least squares (2sls) and an extended regression model (ERM) to deal with the endogeneity problem. Happiness can be considered as both continuous and ordered variables. The two-stage least squares method solves the problem of two-way effects of the independent and dependent variables through two linear regressions, which is applicable when the dependent variable is continuous. The extended regression model (ERM) is based on the multivariate normal distribution and great likelihood estimation. It has the advantage of handling endogeneity problems with endogenous variables, endogenous sample selection, random effects, or a combination of scenarios simultaneously (Liu et al., 2021). The extended model under the ERM framework contains the eregress, eoprobit submodule. Considering happiness as an ordered variable, the extended ordered probit (eoprobit) model in the ERM framework is applied to deal with endogeneity and as a robust regression.

### Data and samples

The data used in this paper originate from the China Family Panel Studies (CFPS) conducted by the China Social Science Research Center of Peking University. The CFPS is conducted every 2 years and provides data from 25 provinces (municipalities directly under the central government) across China. The database contains individual-level and household economic information, which can comprehensively and systematically reflect the characteristics of Chinese micro subjects. These data provide a good database for this study. This paper discusses the relationship between digital literacy and happiness based on the digital economy. In theory, it needs to be studied in a perfect information and communication facilities environment. In 2018, the scale of China’s digital economy accounted for 34.8% of the total GDP, and the Internet penetration rate was 59.6%. The current construction of information and communication facilities in China can be considered as relatively advanced, and the digital environment has gradually formed. Therefore, this paper uses the latest data from CFPS (2018) as the main data source to test the impact of digital literacy on subjective happiness. The variables of digital literacy, happiness, and individual characteristics are derived from the adult pool, and the variables of income and population size are derived from the family pool. The sample screening process is described in the following.

First, respondents aged 15–64 who have an agricultural household and are of working age were selected for this study. All samples outside of this age group and those from non-rural areas were excluded. Second, low-income groups were screen out, relying on the income data of the previous period (CFPS2016) to identify low-income groups. The reason is that the current period income (CFPS2018) may result from the generation of digital literacy, making it difficult to clearly identify the study population. In this paper, the relative poverty criteria set by the World Bank are used. The average income proportion method is used to measure the low-income group, and households with a *per capita* net income below 50% of the average income are considered as low-income households. Finally, exclusion of samples with abnormal or missing core variables formed a sample of 3,013.

### Variables

#### Subjective happiness

In this paper, the dependent variable is the subjective happiness of individuals in rural low-income households. Following the previous literature (Oishi et al., 2011; Ma et al., 2020; Qiu et al., 2021), a “self-rated subjective level of happiness” was used to measure happiness. The CFPS asks each respondent, “How happy are you?” The respondents were asked to express their subjective happiness through a range of scores from 0 (lowest happiness) to 10 (highest happiness).

#### Digital literacy

In this paper, the independent variable is digital literacy. Digital literacy is defined as an individual’s ability to use digital tools and devices correctly and rationally to utilize digital resources, learn new knowledge, and communicate socially with others (DiMaggio and Bonikowski, 2008; Shan et al., 2022). Therefore, the identification of digital literacy is divided into two steps: first, it was determined whether the respondent had the hardware needed for using digital tools and devices based on whether he or she used a device such as a computer or a cell phone to access the Internet. If the answer is no, a value of 0 is assigned. Second, the level of digital literacy of individuals is calculated. According to the realization scenarios of digital literacy, digital literacy reflects the individual’s ability to use digital technology, which typically contains multiple functions, such as learning, working, socializing, entertainment, and business activities. Digital literacy is assigned based on how often individuals use different digital functions, including how often they use the Internet for learning, working, socializing, entertainment, and business activities. The frequency of the use of digital functions ranged from “never” to “almost every day” on a scale of 1–7, respectively. Finally, to obtain the digital literacy level of individuals, the frequency of using different digital functions is calculated by the equal weighting method.

#### Control variables

This paper also controls for other factors that may affect happiness, including common individual and household characteristics, such as age, gender, marital status, health status, political identity, religious beliefs, and educational attainment. Among these, individual age may have a non-linear effect on happiness. Therefore, both individual age and the squared term of age are introduced; household characteristics mainly control for household size and household *per capita* income. The influence of unobservable factors at the village level is considered (e.g., village-specific cultural climate) and village dummy variables are controlled for.

The problem of omitted variables because of possible non-observables is also considered, as it can cause bias in the estimation results. Based on the full coverage of economics, sociology, and psychology, refer to the study of Stead and Bibby (2017), the “Big Five” personality classification is used to identify the personality characteristics of individuals. The CFPS 2018 contained three sub-questions for each personality trait, and the answers ranged from not at all to fully conforming to five levels. In this paper, “talkative,” “outgoing and social,” and “reserved” were used reflect extroversion, while “serious,” “efficient,” and “often lazy” were used to reflect conscientiousness. “Tolerant by nature,” “sometimes rude to others,” and “considerate of others” were used to reflect affinity. “Original and generates new ideas,” “values artistic and aesthetic experiences,” and “imaginative” were used to reflect openness. “Worries a lot,” “gets nervous easily,” and “copes well with stress” were used to reflect emotional stability. The results of the respondents’ responses were reassigned to make indicators internally consistent, as they may have opposite meanings.

### Descriptive statistics

Table 1 presents the results of variable definitions, assignments, and descriptive statistics. Currently, the mean value of digital literacy in rural low-income groups in China is only 1.509 points, which is relatively low. Digital social and entertainment literacy are higher than other dimensions of digital literacy. The mean happiness score for the rural low-income group was 7.278, and a happiness score of 7.486 was calculated for the non-low-income group. The subjective happiness of the low-income group was slightly lower than that of the non-low-income group. Therefore, the subjective happiness of the low-income group requires further attention.

**Table 1:** Summary statistics.

	Variable	Description	*N*	Mean	S.D.
Dependent variable	Happiness	Evaluation of subjective happiness: 1–10 scores	3,013	7.289	2.274
Independent variable	Digital literacy	Multi-indicator calculation: 1–7 scores	3,013	1.509	2.006
Individual characteristics	Age	Age of a person in the sample	3,013	44.18	13.34
	Age squared	Age squared divided by 100	3,013	21.30	11.31
	Gender	Male = 1, otherwise = 0	3,013	0.462	0.499
	Marital status	Married = 1, otherwise = 0	3,013	0.825	0.380
	Health	Self-assessed health: 1–5 scores	3,013	2.910	1.341
	Political identity	Communist party member = 1, otherwise = 0	3,013	0.005	0.073
	Religious beliefs	Religious = 1, otherwise = 0	3,013	0.024	0.154
	Education	Maximum number of years of education	3,013	6.124	4.531
Family Characteristics	Family size	Number of family members (persons)	3,013	4.932	2.235
	Household income	Household net income *per capita* (yuan)	3,013	8.884	0.849
Personality trait	Extroversion	Evaluation of extroversion: 1–5 scores	3,013	3.321	0.705
	Due diligence	Evaluation of due diligence: 1–5 scores	3,013	3.836	0.662
	Affinity	Evaluation of affinity: 1–5 scores	3,013	3.784	0.620
	Openness	Evaluation of openness: 1–5 scores	3,013	3.135	0.903
	Emotional stability	Evaluation of emotional stability: 1–5 scores	3,013	2.910	0.708

When an individual’s marital status is first marriage, in marriage, or cohabitation, he or she is considered as married; if the marital status is unmarried, divorced, or widowed, he or she is considered as single.

**Figure 1:**
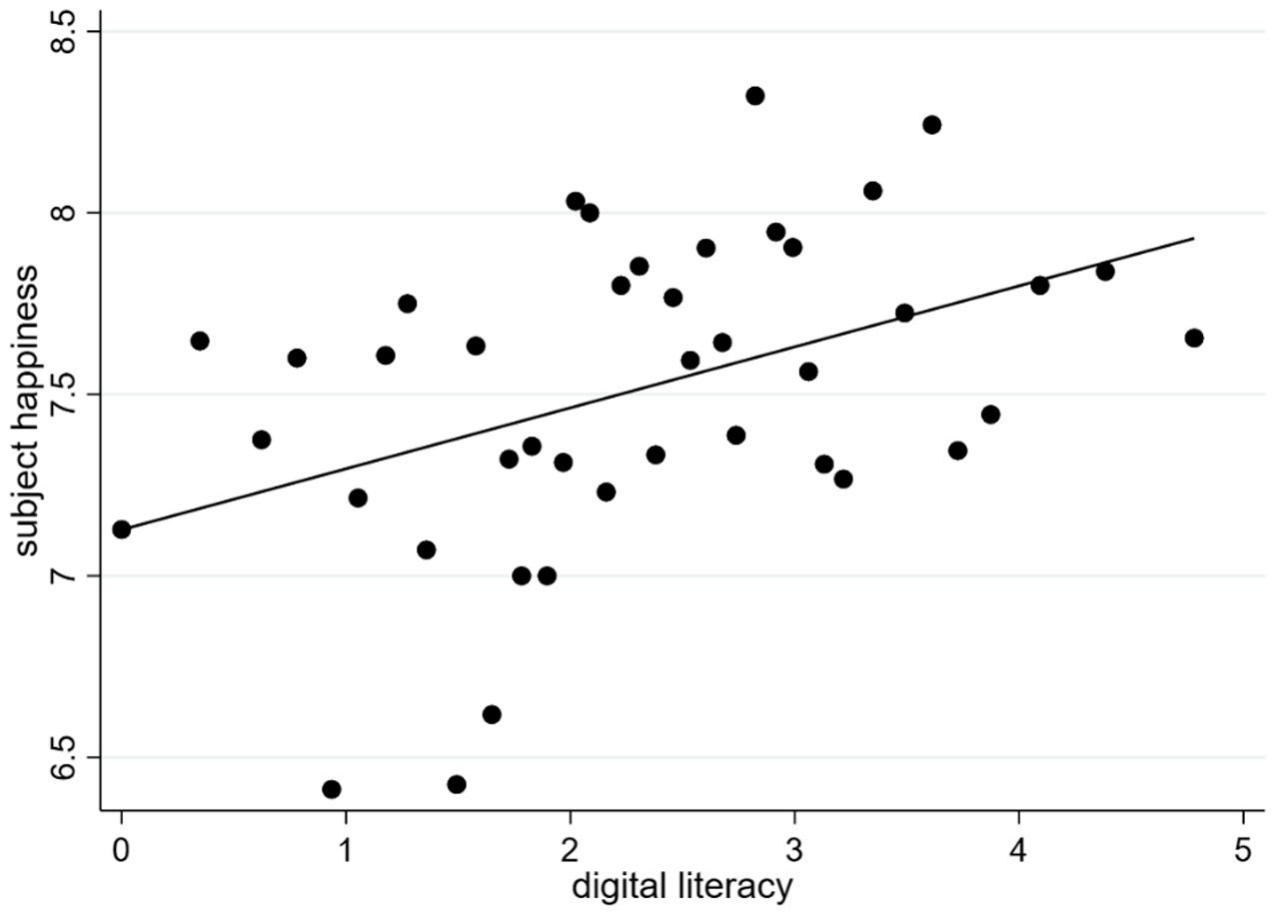
Distribution of the relationship between digital literacy and subjective happiness.

Figure 1 shows the distribution of digital literacy and subjective happiness in rural low-income groups based on the above-mentioned variables and data. Using a bin scatter plot, the relationship between digital literacy and the happiness of rural low-income groups is fitted. The number of bins defaulted to 100, and the black dots in the plot indicate the mean values of digital literacy and subjective happiness in each bin. The slope of the fitted curve is greater than 0. As digital literacy increases, the subjective happiness of the rural low-income group also increases. Therefore, a positive relationship exists between digital literacy and the subjective happiness of rural low-income groups.

## Results

### Baseline results

Table 2 reports the results of baseline regressions. In the column (1), which controls for basic variables, digital literacy, individual characteristics, and family characteristics were added as control variables. Personality trait variables and village dummy variables are arranged in the column (2) on top of the first column to alleviate estimation bias caused by omitted variables. The estimation results using ols show that digital literacy has a significant positive effect on the happiness of rural low-income groups at the 1% statistical level. This indicates that enhancing digital literacy helps to enhance the subjective happiness of rural residents. In an economic sense, the marginal effect of digital literacy is 0.066, and currently, the mean value of digital literacy in the low-income group is 1.509. If the digital literacy of rural residents were to increase by one standard deviation, their subjective happiness would increase by 0.132 points. In this paper, subjective happiness is considered an ordered response variable that takes a value of 1–10. To test the robustness of the regression results, the oprobit model is used for the regression, and the column (3) and column (4) of Table 2 show the estimation results. Increasing the digital literacy of rural low-income groups positively affects their subjective happiness.

**Table 2:** Estimated results of the effect of digital literacy on the happiness of rural low-income groups.

Variables	Subjective happiness			
	(1)	(2)	(3)	(4)
Digital literacy	0.078^***^ (0.025)	0.066^***^ (0.025)	0.033^***^ (0.012)	0.028^**^ (0.012)
Age	−0.089^***^(0.024)	−0.083^***^(0.024)	−0.038^***^ (0.011)	−0.035^***^(0.012)
Age squared	0.113^***^ (0.027)	0.101^***^ (0.027)	0.050^***^ (0.013)	0.044^***^ (0.013)
Gender	−0.138 (0.083)	−0.164^*^ (0.085)	−0.061 (0.039)	−0.074^*^ (0.041)
Marital status	0.325^**^ (0.143)	0.280^*^ (0.144)	0.157^**^ (0.067)	0.138^**^ (0.068)
Health	0.342^***^ (0.033)	0.307^***^ (0.034)	0.163^***^ (0.016)	0.149^***^ (0.017)
Political identity	−0.777 (0.495)	−0.836 (0.541)	−0.356^*^ (0.212)	−0.368 (0.236)
Religious beliefs	0.032 (0.239)	−0.045 (0.233)	0.002 (0.115)	−0.034 (0.114)
Education	−0.002 (0.011)	−0.004 (0.011)	−0.003 (0.005)	−0.004 (0.005)
Family size	0.048^***^ (0.019)	0.045^**^ (0.019)	0.021^***^ (0.009)	0.020^**^ (0.009)
Household income	0.129^***^ (0.049)	0.101^**^ (0.051)	0.059^**^ (0.023)	0.047^*^ (0.024)
Extroversion		0.233^***^ (0.061)		0.116^***^ (0.030)
Due diligence		0.052 (0.069)		0.025 (0.033)
Affinity		0.355^***^ (0.070)		0.172^***^ (0.034)
Openness		0.061 (0.049)		0.029 (0.024)
Emotional stability		0.153^***^ (0.060)		0.069^**^ (0.029)
Village features		Control		Control
*R*-squared	0.059	0.080	0.015	0.021
*F*-value	16.61^***^	15.37^***^		
Wald Chi^2^			156.99^***^	223.45***
Observations	3,013	3,013	3,013	3,013

Robust standard errors are shown in parentheses. ^***^, ^**^, and ^*^ denote the significance of regression coefficients at the 1, 5, and 10% levels, respectively.

The existing literature is more concerned with the impact of ICT on happiness and focuses on the relationship between changes in the digital environment and happiness (Long et al., 2020). This paper focuses on the effect of digital human capital of individuals in the digital environment on happiness. The empirical results of this paper are largely consistent with the above literature, namely, digital literacy has significant information welfare effects, such as increasing online interactions among individuals, reducing information asymmetry, releasing consumption potential and optimizing consumption structure, which in turn increase individuals’ happiness (Purcell et al., 2013; Yang et al., 2021).

2sls and ERM were used for parameter estimation. Table 3 shows the estimation results, where the column (1) and column (2) are the two-stage regression results of 2sls, and the column (3) and column (4) are the two-stage regression results of ERM. In the 2sls estimation results, the DWH test showed that the original hypothesis of digital literacy as an exogenous variable was not rejected. The *F*-value of the one-stage estimation is 231.37, indicating that there is no weak instrumental variable problem. In the ERM estimation results, the residual term correlation coefficient is significant, indicating that the results of the instrumental variable method are more reliable than the baseline regression. Overall, when the endogeneity issue has been dealt with, digital literacy significantly enhances the subjective happiness of the rural low-income group at the 1% statistical level. This result supports Hypothesis 1.

**Table 3:** Estimation results of the instrumental variable method.

Variables	**2sls**		**ERM**	
	**First stage**	**Second stage**	**First stage**	**Second stage**
	(1)	(2)	(3)	(4)
Digital Literacy		0.302^***^ (0.068)		0.088^***^ (0.019)
Tool variables	0.457^***^ (0.021)		0.777^***^ (0.018)	
Control variables	Control	Control	Control	Control
*F*-test	231.37^***^		231.37^***^	
DWH test		14.348^***^		
Correlation coefficient of the residual term				−0.135^***^ (0.031)
Observations	3,013	3,013	3,013	3,013

Robust standard errors are shown in parentheses. ^***^, ^**^, and ^*^ denote the significance of regression coefficients at the 1, 5, and 10% levels, respectively. The Durbin–Wu–Hausman test (DWH test) is usually used to test whether the independent variables are endogenous.

### Robustness test

The robustness of the baseline regression results is tested by replacing the core independent variable, the dependent variable, and the health variable.

First, the core independent variables are replaced. In this paper, principal component analysis is used to calculate digital literacy (Zhao and Li, 2021). The principal component analysis model contains five indicators, and a total of one common factor is extracted using the principal component method. A Kaiser–Meyer–Olkin (KMO) value of 0.858 (> 0.7) and a significant *p* value of 0.000 for Bartlett’s test were obtained, indicating a strong correlation between indicators and the results of reliable factor analysis. The cumulative variance contribution was 73.34%, indicating that this common factor has relatively strong explanatory power for the indicators. The column (1) of Table 4 presents the estimation results of replacement digital literacy variables. The digital literacy obtained from the principal component analysis statistically positively affects the subjective happiness of the rural low-income group at the 1% level, which is consistent with the baseline regression model.

**Table 4:** Results of robustness tests.

Variables	Subjective happiness		
	(1)	(2)	(3)
New digital literacy	0.144^***^ (0.049)		
Digital literacy		0.045^***^ (0.016)	0.065^***^ (0.025)
Control variables	Control	Control	Control
Mental health			Control
*R*-squared	0.079	0.047	0.112
*F*-value	16.15^***^		21.79^***^
Wald Chi^2^		183.04^***^	
Observations	3,013	2,858	3,013

Robust standard errors are shown in parentheses. ^***^, ^**^, and ^*^ denote the significance of regression coefficients at the 1, 5, and 10% levels, respectively.

Second, the dependent variable was replaced. The dummy variable for happiness was adjusted according to the mean, and values greater than the mean were set to 1 (Long et al., 2020). Regression analysis was conducted using the Probit model, and the column (2) of Table 4 shows the estimated results after replacement of the dependent variable. After this replacement, digital literacy still has a significant positive effect on the subjective happiness of the rural low-income group. This further evidence also supports the happiness effect of digital literacy.

Third, the individual health measure variable is replaced. Happiness is an emotional experience of real life that is closely related to the psychological state of an individual (Ford et al., 2015). If an individual is psychologically depressed, it is difficult to achieve a high level of happiness even if the individual does not live in a state of relative deprivation. Therefore, the CFPS database measures the mental health of individuals with the help of questions on depression and related cognitive aspects. The depression level is how often the respondent experienced negative emotional or physical adverse reactions over the past week, such as low mood, poor sleep, and feeling lonely. Respondents’ depression scores were obtained by summing problem scores, where higher scores indicate more severe depression. Mental health variables replaced self-rated health variables in the regression equation, and the column (3) of Table 4 demonstrates the estimation results after replacing the individual health measure approach. The results show that digital literacy significantly contributes to the subjective happiness of individuals in the rural low-income group. This reflects the significant presence of the happiness effect of digital literacy and demonstrates the robustness of the study results.

### Heterogeneity analysis

Overall, digital literacy positively affects the subjective happiness of rural low-income groups. However, the results may differ for groups with different characteristics. To clarify the effect of digital literacy on the subjective happiness of rural low-income groups with different characteristics, heterogeneity analysis was conducted in terms of both education level and poverty level.

First, rural low-income groups were classified into low education (less than six years) and high education (more than six years) according to their number of years of education (Li et al., 2017). The column (1) and column (2) of Table 5 show the results of subgroup regressions. Digital literacy significantly enhances the subjective happiness of low-income individuals in the low-education group. However, the effects on the happiness of low-income and high-education groups are not significant. Because education and digital literacy belong to human capital, human capital accumulation is subject to a significant law of diminishing margins. High levels of educational attainment may crowd out the role of digital literacy in increasing the happiness of low-income groups.

**Table 5:** Estimation results of group regression.

Variables	Low education	High education	Deep poverty	Non-deep poverty
	(1)	(2)	(3)	(4)
Digital literacy	0.100^**^	0.045	0.104^*^	0.069^**^
	−0.047	−0.029	−0.058	−0.026
Control variables	Control	Control	Control	Control
*R*-squared	0.041	0.105	0.059	0.084
*F*-value	6.90^***^	10.82^***^	4.08^***^	14.54^***^
Observations	1,562	1,451	613	2,400

Robust standard errors are shown in parentheses. ^***^, ^**^, and ^*^ denote the significance of regression coefficients at the 1, 5, and 10% levels, respectively.

Rural low-income groups are further classified into deep poverty and non-deep poverty groups based on the *per capita* income of households. As the basis for this classification, the poverty line (¥ 2,300) set by the Central Poverty Alleviation Work Conference at the end of 2011 was used (Wan et al., 2021). If a farmer’s income is below this poverty line, this farmer was considered to be deeply poor. If the farmer’s income exceeds this poverty threshold, the farmer is considered as non-deeply poor. The column (3) and column (4) of Table 5 show the results of subgroup regressions. The results show that digital literacy positively affects the subjective happiness of both the deeply poor and the non-deeply poor in the rural low-income group. However, the effect on the subjective happiness of the deeply poor rural low-income group exceeds that of deeply poor households. This result suggests that digital literacy reflects a marginal decreasing rather than a scale-increasing characteristic across income levels. The reason may be that digital literacy is a form of human capital that enhances endogenous motivation, while income is an epiphenomenon of ability. Deeply poor people with relatively low ability can quickly embed in the new form of the digital economy after enhancing their digital literacy, narrowing the gap to other groups, and thus alleviating the sense of deprivation compared with others.

Some scholars have found that Internet use has a depressing effect on the happiness of low-literacy groups (Yang et al., 2021). However, we found that digital literacy has a facilitative effect on the happiness of low literacy groups, which is consistent with the fact that human capital accumulation has a marginal decreasing law. Moreover, digital literacy is also associated with digital resilience, the capacity of individuals to make effective use of digital information to prevent, anticipate, absorb and adapt to major challenges, and to continuously evolve and transform in a productive and sustainable manner (Tim et al., 2021). This ability contributes to subjective happiness. The empirical finding that digital literacy is marginal decreasing rather than increasing in size across income levels is consistent with the existing literature (Shan et al., 2022). These results side-by-side reflect that digital literacy helps to reduce the happiness gap between different education and income groups in rural areas, thus increasing the happiness of the overall rural low-income group.

### Impact mechanism test

The results of the previous empirical analysis indicate that the enhancement of digital literacy can significantly contribute to the happiness of rural low-income groups. The question remains through which influence mechanism digital literacy affects the happiness of rural low-income groups. Based on the relative deprivation theory, this paper identifies two impact mechanisms: the income effect and the consumption effect. Therefore, this section tests whether the income effect and the consumption effect of digital literacy exist.

#### Test of the income effect of digital literacy

Scholars agree that absolute and relative incomes significantly affect happiness in the income-happiness relationship. Already in Easterlin (1974), argued that there is no absolute linear relationship between income and happiness but that it is an inverted U-shaped relationship. When income reaches a certain threshold, happiness decreases as income increases. However, the income level of rural low-income groups remains below this inflection point; therefore, in low-income groups, the relationship between income and happiness is very likely linear.

In this paper, the linear relationship between digital literacy and income is discussed from the two perspectives of absolute and relative income. Absolute income uses the current net *per capita* and takes the logarithmic form. Relative income uses a commonly used subjective measure (Graham and Pettinato, 2001): the level at which respondents perceive their income compared to others. In the CFPS database, the question “How much do you earn locally?” was rated from very low to very high on a scale of 1–5. In the regression model, absolute and relative incomes were substituted as dependent variables. It is important to note that there is likely an inverse causal relationship between income and digital literacy; therefore, the regression was conducted using the least squares estimation and instrumental variables method. Table 6 presents the estimation results, where the column (1) and column (3) are estimated using ols, the column (2) and column (4) are estimated using 2sls.

**Table 6:** Estimates of the impact of digital literacy on absolute and relative income.

Variables	Absolute income		Relative income	
	(1)	(2)	(4)	(4)
Digital literacy	0.068^***^ (0.010)	0.083^***^ (0.026)	−0.001 (0.013)	0.126^***^ (0.038)
Control variables	Control	Control	Control	Control
F-value	16.99^***^		12.04^***^	
Wald chi2		215.86^***^		181.44^***^
DWH test		0.374		14.350^***^
R-squared	0.079	0.078	0.067	0.042
Observations	3,013	3,013	2,851	2,851

Robust standard errors are shown in parentheses. ^***^, ^**^, and ^*^ denote significant regression coefficients at the 1, 5, and 10% levels, respectively. The first stage estimation results of 2sls are not shown because of space limitation, but all models pass the weak instrumental variables test.

First, the relationship between digital literacy and absolute income is assessed. The results in the column (2) of Table 6 show that the model does not pass the DWH test, which is interpreted regarding the ols results. The results in the column (1) show that digital literacy statistically positively affects absolute income at the 1% level, suggesting that digital literacy promotes absolute income growth among rural low-income groups. Second, the relationship between digital literacy and relative income is assessed. The results in the column (4) show that the model passes the DWH test and the original hypothesis that digital literacy is an exogenous variable is rejected. This is interpreted regarding the estimation results of the 2sls. As shown in the column (4), digital literacy statistically positively affects relative income at the 1% level, indicating that digital literacy alleviates the relative income gap between rural low-income groups and other groups.

This empirical result is consistent with the theoretical analysis and existing literature findings. Individuals are processors of information, and the improvement of digital literacy can better exploit the information and employment effects of digital technology, thus contributing to an increase in absolute income (Ma et al., 2020). The digital economy is inclusive in nature. Digital literacy helps low-income groups to participate more adaptively, rationally, and effectively in digitally relevant areas of production and life, thereby alleviating income inequality and narrowing the relative income gap (Kuhn and Mansour, 2014; Philip et al., 2017). The increase in absolute income and the reduction in relative income gap led to an increase in subjective well-being of rural low-income groups.

#### Test of the consumption growth effect of digital literacy

The authors argue that digital literacy may help rural low-income groups pursue social and economic rights. The relationship between digital literacy and consumption growth is further tested using the consumption structure to reflect the reality of the implementation of rights. In the consumption-happiness relationship, expenditures on food, housing, health services, and education contribute to subjective happiness. For low-income households, meeting the subsistence needs and alleviating the relative deprivation level in the right to development and enjoyment can greatly enhance their subjective happiness. Refer to the division of consumption structure by Qi and Liu (2020), in the present paper, the consumption growth effect of digital literacy is analyzed from the three aspects of survival consumption, developmental consumption, and enjoyment consumption. Among the forms of consumption, survival consumption includes expenditure on food, housing, clothing, and footwear; development consumption includes household equipment, daily necessities, health care, transportation, and communication consumption; enjoyment consumption includes expenditure on culture, education, and entertainment. Considering the potential reverse causality between consumption and digital literacy, the parameters were estimated using least squares and instrumental variables methods, respectively. Considering the possible reverse causality between consumption and digital literacy, least squares and instrumental variables methods were used for parameter estimation, respectively. The column (1), column (4), and column (5) of Table 7 show the estimation results of the ols. The column (2), column (4), and column (6) of Table 7 show the estimation results of 2sls.

**Table 7:** Estimation results of how digital literacy affects consumption structure.

Variables	Survival consumption		Developmental consumption		Enjoyable consumption	
	(1)	(2)	(3)	(4)	(5)	(6)
Digital literacy	0.040^***^	0.01	0.153^***^	0.255^***^	0.504^***^	0.109^***^
	−0.01	−0.024	−0.038	−0.079	−0.012	−0.028
Control variables	Control	Control	Control	Control	Control	Control
F-value	18.21^***^		39.27^***^		19.46^***^	
Wald chi2		383.18^***^		569.47^***^		369.76^***^
DWH test		0.467		2.882^*^		7.598^***^
R-squared	0.104	0.148	0.173	0.173	0.11	0.124
Observations	2,938	2,938	2,979	2,979	2,890	2,890

Robust standard errors are shown in parentheses. ^***^, ^**^, and ^*^ denote significant regression coefficients at the 1, 5, and 10% levels, respectively. The first stage estimation results of 2sls are not shown because of space limitation, but all models pass the weak instrumental variables test.

First, the relationship between digital literacy and survival-oriented consumption is analyzed. The results in the column (2) show that the model does not pass the DWH test, which is interpreted as resulting from the results of ols. As shown in the column (1), digital literacy statistically positively affects survival-oriented consumption at the 1% level, indicating that digital literacy promotes the growth of survival-oriented consumption among rural low-income groups. Second, the relationship between digital literacy and developmental consumption is observed. The results in the column (4) show that the model passes the DWH test and the original hypothesis that digital literacy is an exogenous variable is rejected. This is interpreted as resulting from the estimation results of the 2sls. The results in the column (4) show that digital literacy statistically positively affects developmental consumption at the 1% level, reflecting that digital literacy promotes the growth of developmental consumption among rural low-income groups. Finally, the relationship between digital literacy and enjoyment-based consumption is assessed. The results in the column (6) show that the model passes the DWH test, which is interpreted as being the result of the estimation results of the 2sls. The results show that digital literacy statistically positively affects enjoyment-based consumption at the 1% level, reflecting that digital literacy raises the enjoyment-based consumption of rural low-income groups.

The findings that digital literacy has consumption effects are consistent with the existing literature. In China, the rise and development of ICT and the technologies, products, and services it has spawned have greatly enriched rural households’ production patterns and lifestyles, such as online shopping, digital education, and platform healthcare (Nishijima et al., 2017). Digital literacy is human capital in the digital environment, which is the ability of individuals to process information (Vuong and Napier, 2015). Digital literacy helps low-income groups reap the basic rights of survival needs and close the relative gap between the development and enjoyment of rights. Because low-income groups can satisfy their survival needs and pursue more rights to development and enjoyment needs, thus enhancing subjective well-being (Weidman and Dunn, 2016).

### Further test

#### Decomposing happiness: Job satisfaction and life satisfaction

As an individual’s emotional response to real society, happiness reflects the individual’s overall experience of his or her life circumstances (including the work environment). Refer to Ilies and Judge (2004), individuals’ cognitive evaluations of their life state were measured in terms of life satisfaction, and their emotional reactions, cognitive evaluations, and affective experiences of their jobs were measured in terms of job satisfaction. Considering the potential endogeneity between digital literacy, job satisfaction, and life satisfaction, regressions were conducted using the ols and 2sls. The column (1) and column (3) of Table 8 show the results of the ols, and the column (2) and column (4) show the 2sls results.

**Table 8:** Estimated results of how digital literacy affects different types of subjective happiness.

Variables	Job satisfaction		Life satisfaction	
	(1)	(2)	(3)	(4)
Digital literacy	0.057^***^	0.125^***^	−0.001	0.120^***^
	−0.017	−0.046	−0.011	−0.029
Control variables	Control	Control	Control	Control
F-value	27.03^***^		13.20^***^	
Wald chi2		437.29^***^		260.70^***^
DWH test		1.421		17.31^***^
R-squared	0.135	0.136	0.062	0.044
Observations	3,013	3,013	3,013	3,013

Robust standard errors are shown in parentheses. ^***^, ^**^, and ^*^ denote significant regression coefficients at the 1, 5, and 10% levels, respectively. The first stage estimation results of 2sls are not shown because of space limitation, but all models pass the weak instrumental variables test.

First, regarding the relationship between digital literacy and job satisfaction, the results in the column (2) of Table 8 show that the model does not pass the DWH test, which is interpreted to result from ols. The results in the column (1) show that digital literacy statistically positively affects job satisfaction among rural low-income groups at the 1% level. Second, regarding the relationship between digital literacy and life satisfaction, the results in the column (4) show that the model passes the DWH test and the original hypothesis that digital literacy is an exogenous variable is rejected. This is interpreted as being the result of the estimation results of 2sls. The results in the column (4) show that digital literacy statistically positively affects life satisfaction at the 1% level.

This empirical result is supported by previous literature. Digital literacy helps low-income groups to search for and find jobs that match their abilities and promote their career mobility to non-agricultural industries (Kuhn and Mansour, 2014), thus increasing job satisfaction. Rural low-income groups can view and purchase goods on digital platforms, thus increasing their life satisfaction through consumption (Holder, 2019). The diversified entertainment functions developed by digital platforms, short videos, and other entertainment applications improve the quality of leisure time for rural low-income groups (Campante et al., 2018).

## Conclusion

Taking the low-income group in rural China as research object, this study explored the influence and mechanism of how individual digital literacy affects the subjective happiness of rural residents. The factors that would play a moderating role in this process of promoting or inhibiting the subjective happiness of this group were investigated. The empirical results show that first, digital literacy has a significant happiness effect on rural low-income groups. Digital literacy helps low-income groups reduce the cost of information seeking, processing and dissemination, thereby increasing subjective happiness. Second, in terms of the mechanism, digital literacy mainly affects subjective happiness low-income groups by increasing household income and promoting household consumption. Third, digital literacy significantly impacts the subjective happiness of rural low-income groups with low education level and who experience deep poverty. Fourth, the decomposition of happiness into life satisfaction and job satisfaction, shows that digital literacy has a significant positive impact on job satisfaction and life satisfaction of rural low-income groups.

## Recommendation

This study also has important practical implications. First, the government should focus on cultivating the digital literacy of low-income groups to effectively provide development and endogenous motivation for enhancing subjective happiness. The government should strengthen the digital education of rural residents and establish a digital literacy cultivation system that meets the needs of rural development. The government can organically play the role of different social organizations in improving the digital literacy of the rural population, for example through school education and social organization training.

Second, the government must continue to promote the construction of the digital village, including digital infrastructure construction and rural industry digitization. The government’s improvement of digital infrastructure construction enables the popularization of the Internet and the digital transformation of transportation, energy, and logistics. For example, an intelligent distribution system is vital for circulating urban and rural commodities, and enables convenient circulation of urban and rural products and services.

## Limitation

The present study has certain limitations. First, this study did not employ a cross-country sample to test the relationship between digital literacy and subjective happiness. Differences exist in the degree of information and communication facilities among different countries, and the digital environment in which rural low-income groups are located may also differ. The role of digital literacy is strongly related to the digital environment; therefore, whether the findings of this paper can also be applied to other countries remains unknown.

Second, the Internet is a platform for information interaction, and the cultural values of individuals affect the process of information processing, which ultimately influences the role digital literacy plays for happiness. However, the data is limited, the cultural values variable cannot be controlled, and it is difficult to observe the heterogeneous influence induced by cultural values. Subject to traditional culture, Chinese villages have a unique cultural atmosphere that also affects the cultural values of villagers. Thus, village-level fixed effects are controlled for and the influence of cultural differences is partially absorbed.

## Data availability statement

The raw data supporting the conclusions of this article will be made available by the authors, without undue reservation.

## Author contributions

JW and CL designed and conducted the study and wrote the manuscript. JW analyzed the data. ZC reviewed and revised the manuscript. All authors contributed to the article and approved the submitted version.

## Funding

This study was supported by grants from the National Natural Science Foundation of China and the Ministry of Education of China (grant no. 72003091 and 17YJA790004).

## Conflict of interest

The authors declare that the research was conducted in the absence of any commercial or financial relationships that could be construed as a potential conflict of interest.

## Publisher’s note

All claims expressed in this article are solely those of the authors and do not necessarily represent those of their affiliated organizations, or those of the publisher, the editors and the reviewers. Any product that may be evaluated in this article, or claim that may be made by its manufacturer, is not guaranteed or endorsed by the publisher.

## Footnotes

^1^The 49th Statistical Report on the Development Status of China’s Internet: By the end of 2021, China’s Internet penetration rate reached 73%, and the rural Internet penetration rate was 57.6%. http://www.cnnic.net.cn/hlwfzyj/hlwxzbg/hlwtjbg/202202/t20220225_71727.htm.
